# T Helper Subsets, Peripheral Plasticity, and the Acute Phase Protein, *α*1-Antitrypsin

**DOI:** 10.1155/2015/184574

**Published:** 2015-10-25

**Authors:** Boris M. Baranovski, Gabriella S. Freixo-Lima, Eli C. Lewis, Peleg Rider

**Affiliations:** Department of Clinical Biochemistry & Pharmacology, Faculty of Health Sciences, Ben-Gurion University of the Negev, 84101 Be'er Sheva, Israel

## Abstract

The traditional model of T helper differentiation describes the naïve T cell as choosing one of several subsets upon stimulation and an added reciprocal inhibition aimed at maintaining the chosen subset. However, to date, evidence is mounting to support the presence of subset plasticity. This is, presumably, aimed at fine-tuning adaptive immune responses according to local signals. Reprograming of cell phenotype is made possible by changes in activation of master transcription factors, employing epigenetic modifications that preserve a flexible mode, permitting a shift between activation and silencing of genes. The acute phase response represents an example of peripheral changes that are critical in modulating T cell responses. *α*1-antitrypsin (AAT) belongs to the acute phase responses and has recently surfaced as a tolerogenic agent in the context of adaptive immune responses. Nonetheless, AAT does not inhibit T cell responses, nor does it shutdown inflammation per se; rather, it appears that AAT targets non-T cell immunocytes towards changing the cytokine environment of T cells, thus promoting a regulatory T cell profile. The present review focuses on this intriguing two-way communication between innate and adaptive entities, a crosstalk that holds important implications on potential therapies for a multitude of immune disorders.

## 1. Introduction

The necessity to defend against a diverse world of pathogens, toxins, or transformed cells requires different types of immune responses in order to overcome a pathological entity while maintaining tolerance towards self-tissues. During evolution, vertebrates developed lymphocytes that bare combinatorial T cell receptors (TCR) and B cell receptors (BCR) in order to overcome the enormous and evolving variety of antigenic events. An accurate adaptive response must accommodate the type of pathogen, its mechanism of intrusion, and the tissue that is affected. The response must be tightly regulated as undesirable collateral damage from autoreactive T cells and antibodies can result in autoimmune disorders and hypersensitivity responses, some as common as allergies. Allograft transplantation is another platform in which the activity of the adaptive immune response is the target of therapeutic manipulation. A rigid determination of the type of immune response, without retaining flexibility or regulation of an activated T cell and an appreciation of the changing environment along the adaptive immune response, can result, on the one hand, in an often lethal organ failure associated with the site of infection, or, on the other hand, promotion of autoimmunity that follows an infection.

Endowing the immune system with local communication capacity through a diverse array of cytokines represents an important mechanism for an environment to control T cell responses. T helper cells initially differentiate according to local cytokine composition, resulting in different subsets of CD4^+^ T helper cells. The composition of cytokines is so influential that even a subtle change in one of the components, such as IL-6 in a TGF*β*-rich environment, can facilitate the differentiation of a T helper (TH) subset from a regulatory T cell to an aggressive TH17 effector cell. On top of this apparent complexity, one should note that, to date, the number of established TH subsets is rising; different helper cells appear to conduct a variety of events, such as regulation or enhancement of inflammation, activation of phagocytes, induction of specific cell migration, stimulation of antibody production, or promotion of class switching in activated B cells.

With such a myriad of TH subsets, it becomes more tangible to appreciate the possibility that each TH subset does not necessarily represent its own final differentiation state and that some plasticity exists based on the immediate environment in which activated TH cells roam. As such, we chose the acute phase response to signify a profound environmental change during an adaptive immune response. Within this state of elevated systemic inflammation, it appears that some proteins exert immunomodulatory activities of which a few are indicative of peripheral TH cell plasticity, switching between subsets of TH cell according to the changing environment. One of these proteins, *α*1-antitrypsin (AAT), has recently been recognized as a tolerogenic agent, changing the course of hazardous adaptive processes (e.g., type 1 autoimmune diabetes) towards active antigen-specific immune tolerance.

In this review, we discuss the various CD4^+^ T helper subsets known to date and the signals required for their development. In addition, innovative reports of reprogramming of already differentiated T helper cells towards different helper subsets, and the mechanism allowing the plasticity of helper cells, are presented.

## 2. A Plethora of Helper T Cell Subsets

### 2.1. Traditional Helper T Cell Subset Classification: TH1/TH2

Distinguishing between TH1 and TH2 subtypes was made possible in the late 70s based on two major cell properties: the property of adhering to nylon wool columns and the ability to help B cells produce antibodies, respectively [1]. Mosmann et al. later expanded on the classic TH1/TH2 model [2] and in 1986 introduced several pivotal cell responses to better represent the molecular polarization of helper T cell subtypes: TH1 cells were described as helper T cells that produce interferon-*γ* (IFN*γ*) and interleukin- (IL-) 2, while TH2 cells were described as helper T cells that secrete B cell stimulating factor 1 (BSF1), later termed IL-4 and identified as the factor responsible for driving B cells to produce IgG1 and IgE.

**Figure 1:**
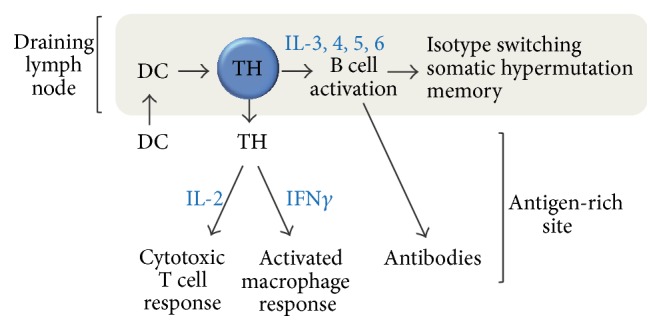
Who do the helper T cells actually help? Once a dendritic cell (DC) activates a helper T cell (TH) in a lymph node that drains an antigenic site, TH can promote B cell responses within the lymph node, as well as circulate the body and relocate to the antigen-rich site for facilitation of cytotoxic T cell responses and local macrophage activation.

The behaviors of TH1 and TH2 in the larger framework of adaptive immune responses were further studied, allowing a more context-dependent classification, that is, the identification of TH1 cells as predominating in the immune response towards* intracellular* pathogens, in which the helper T cell will promote other local T cell responses by secreting IL-2 and activate local macrophages by releasing IFN*γ*, and the identification of TH2 cells as predominating in the immune response towards* extracellular* pathogens, such as helminths, by using a particular set of B cell-activating cytokines and promoting the production of clonal antibodies, such that the antibodies will target the pathogens and either neutralize them, dock the complement system onto them, or mediate local degranulation of mast cells and eosinophils. In this regard, unlike cytotoxic T cells, helper T cells never directly* kill* a target but rather activate local destructive macrophages, drive B cell processes towards an effector humoral response, and fuel neighboring cytotoxic T cells with IL-2 once both these cells become locally attracted to an antigen-rich site (illustrated in Figure 1).

Polarization of TH0 towards TH1/TH2 cells occurs following the exposure of TH0 cell to distinct sets of cytokines in its immediate environment. These cytokines originate primarily from professional antigen presenting cells (pAPCs). pAPCs that encounter a pathogen and engulf related antigens stimulate T cells by forming a TCR-MHC class II complex, with the provision that costimulatory signals are also satisfied; then, particular sets of cytokines may be produced so as to divert the course of T cell differentiation towards either TH1 or TH2.

The major factor that promotes differentiation of TH0 towards TH1 is the dimeric cytokine, IL-12. A lack in the subunit IL-12p40 results in impaired TH1 responses and in an increased susceptibility to intracellular pathogens, such as* Leishmania major* [3, 4]. In contrast, the major cytokine for the differentiation of TH0 into TH2 is IL-4, which will induce the release of IL-5 and IL-13, as well as additional IL-4 [5]. These TH2 cytokines stimulate B lymphocytes towards further maturation, antibody isotype switching and production, somatic hypermutation, and a memory phenotype. In addition, the cytokines will initiate intracellular signals that will induce transcription of genes, which will execute and maintain the consequential T helper subset programming [6]. A transcription factor that is activated downstream to the TCR signal nuclear factor of activated T cells (NFAT) has the ability to bind to either* infg* or* il4* promoters, committing the cell to either TH1 or TH2 phenotype [7]. Additional intracellular signaling pathways activate one of two master transcription factors, either T-bet or GATA-3, which will further consolidate the T helper fate towards being either TH1 or TH2, respectively.

How are these signaling pathway distinctions made? Two signal pathways activate the TH1 transcription factor, T-bet. Following activation of the IL-12 receptor (IL-12R), STAT4 is activated and T-bet is upregulated [8, 9]. T-bet, in turn, activates the transcription of IL-12R*β*2 and IFN*γ*, which then activate STAT1 that also upregulates the transcription of T-bet. This sequence of events enhances additional IFN*γ* transcription, completing a TH1 differentiation positive feedback loop [10]. T-bet acts in synergy with RUNX3 in order to activate IFN*γ* production but at the same time inhibits IL-4 transcription; reciprocity between TH1 and TH2 phenotypes is thus achieved [11]. In contrast, for the differentiation of TH0 into TH2, IL-4R signaling activates STAT6, which upregulates the transcription of GATA-3 [12–14]. GATA-3 then activates the transcription of IL-5 and IL-13; IL-4 production requires the activation of c-Maf [15], which is activated either by GATA-3 or by the TCR signal itself. Thus, GATA-3, in TH2, and T-bet, in TH1, achieve an obligatory reciprocal effect, which is strengthened both by auto-positive-feedback loops and by reciprocal inhibition of the opposing components [3, 9, 14, 16].

### 2.2. A Third Type of Helper T Cell Emerges: TH17

Initially, TH17 cells were termed “IL-23-derived autoreactive CD4 T cells” [17]; subsequently, they were identified as “IL-17-producing T helper cells” [18] and then, finally, TH17 cells [19]. The definition of TH17 lineage had followed the discovery of the cytokine family, IL-17, initially coined CTLA-8 family of cytokines [20, 21]. To date, IL-17 is a common name used to describe the IL-17 family of cytokines, of which the most studied ones are IL-17A and IL-17F; these two share the highest homology amongst the family members and can form a homo- or heterodimer that binds to a single IL-17 receptor (IL-17R) [22]. While the levels of IL-17 and IL-17R correlate with enhanced antimicrobial responses, as observed primarily in mucosal tissues (where IL-17 was found to be secreted from *γδ*T cells [23]), the involvement of IL-17 was also exemplified in several autoimmune disorders [24]. In fact, in as far as autoimmune disorders are regarded, TH17 cells were shown to be involved in some pathologies that were, until then, considered as TH1-related. Examples include EAE, rheumatoid arthritis, and lupus, colitis, in addition to other unwanted immune responses such as acute allograft rejection [17, 25–27].

Interestingly, the distinction between TH1 and TH17 systems was initially made possible by the more in-depth characterization of IL-23 and IL-12. Both these cytokine share the same IL-12p40 subunit. This subunit was targeted, appropriately at the time, in order to study TH1 responses; however, when the individual unique adjoining subunits of IL-12 and IL-23 (IL-12p35 and IL-23p19, resp.) were deleted in mouse genomes, the distinct involvement of TH1 and TH17 cells in autoimmune disorders was unearthed [17, 26]. Nonetheless, it later became apparent that IL-23 alone is not sufficient in order to induce differentiation of TH0 cells towards TH17 cells. It appears that the combination of IL-6 or IL-21, together with TGF*β*, is required in order to advance a TH17 phenotype. In addition, IL-1*β* and TNF*α* exert an amplifying effect on the TH17 cell population, while IL-23 was found to be primarily required for the survival of the already differentiated TH17 subset [28, 29].

Differentiation into TH17 cells requires a master transcription factor, the orphan nuclear receptor, ROR*γ*T (RORC, in humans), or ROR*α*, which also drives the cells toward a TH17 phenotype [30]. While transcription of ROR*γ*T was shown to be dependent on STAT3, which is activated by IL-23 [31], the mechanism by which ROR*γ*T and ROR*α* induce TH17-related genes has yet to be determined.

### 2.3. Regulatory Helper T Cells

In order to regulate the effector arm of T cells, for example, so as to avoid collateral damage or discourage an autoimmune response, two major mechanisms had evolved:* central tolerance*, in which putative T cell clones that recognize self-antigens undergo apoptosis in the thymus before reaching secondary lymphoid organs, and* peripheral tolerance*, in which an effector T cell response is counteracted at the time and location of its activation, based on local factors.

For central tolerance, naturally occurring regulatory T cells (nTreg) develop in the thymus, and their specific depletion results in autoimmune syndromes [32]. Indeed, this subtype was discovered based on the observation that neonatal thymectomy results in autoimmune disorders [33, 34], and reconstitution of CD4^+^ T cells cancels that phenomenon [35, 36]. These cells are characterized by the expression of surface CD4 and, without an apparent need for activation, surface IL-2R*α* (CD25). The source of IL-2, required for Tregs, is the activated effector T cell. Therefore, counter regulation of excessive T cell activities actually increases with T cell activation and clonal expansion, as IL-2 levels rise [37]. The dependence of immune regulation on an intact thymus implies that there exists a development of two different CD4^+^ T cell entities in the thymus: effector CD4^+^ T cells, among which might persist some autoreactive clones that had escaped central tolerance negative selection, and CD4^+^ regulatory T cells, capable of inhibiting the effector CD4^+^ T cells in the periphery.

Controlling the regulatory profile of Tregs, foxp3 was shown to be the major transcription factor of these CD4^+^CD25^+^ T cells. Foxp3 was initially identified as essential for prevention of autoimmune diseases. Indeed, in mice, mutated foxp3 is associated with unregulated T cell activities, and, in humans, mutations in foxp3 correlate with an X-linked syndrome comprised of immunodeficiency polyendocrinopathy and enteropathy (IPEX) [24, 38]. Up to the time of these observations, foxp3 was considered unique to nTreg cells; however, ectopic expression of foxp3 was found to be able to* drive* non-Treg CD4^+^ cells toward a Treg phenotype [39], and extrathymic elevated levels of TGF*β* or retinoic acid were found to induce foxp3^+^ T cells. Thus, such T cells were termed inducible Tregs (iTregs). Importantly, TGF*β* has been shown to require that the rest of the local cytokine milieu does not contain excessive amounts of IL-1*β* or IL-6.

Among the mechanisms in which Tregs can suppress T effector cells are the regulation of APC stimulatory molecules, for example, by expressing CTLA4 that will compromise CD28 signaling, by induction of T cell apoptosis, by deprivation of IL-2, and by secretion of inhibitory cytokines, such as IL-10 and TGF*β*, which in turn stimulate foxp3 expression in T cells [40].

Unlike Tregs, which display the classical CD4^+^CD25^+^foxp3^+^ phenotype, other types of immunosuppressive T cells were described. Two such cell types can have immunoregulatory functions comparable to those mediated by Tregs and are termed TH3 and regulatory T-1 (Tr1) cells. TH3 cells were suggested to induce tolerance following oral administration of antigens to mice. These cells mediate suppression by releasing large amounts of TGF*β*, which in turn can promote regulation by promoting the expansion of iTregs [41]. Tr1 cells, on the other hand, were described as IL-10 producing T cells and, much like foxp3-positive Tregs, they suppress effector T cell proliferation [42]. Differentiation of Tr1 is mediated by IL-10, and the expansion of the population requires IL-27 [43]. The significance of being able to produce IL-10 as a component of T cell suppression was clarified by patients with severe combined immunodeficiency, which exhibited high levels of IL-10-producing donor-derived T cell clones that had mediated tolerance and prevented graft versus host disease (GvHD), despite mismatched HLA and the presence of host-reactive T cells. This phenomenon indicates that peripheral tolerance may be maintained by these IL-10-producing T suppressor cells [44]. In addition, Tr1 cells can be antigen-specific, and reduction in the levels of Tr1 cells can result in autoimmune disorders, such as pemphigus vulgaris, type 1 autoimmune diabetes, rheumatoid arthritis, and autoimmune hemolytic anemia [45]. Moreover, adoptive transfer of Tr1 cells has been shown to promote tolerance in the context of autoimmune disorders and during islet allograft transplantation [46]. While much of the suppression exerted by Tr1 cells relates to IL-10 release [47], they can, like TH3, also secrete TGF*β*. Therefore, Tr1 and TH3 were suggested to promote tolerance by manner of synergism with iTregs [45].

### 2.4. Non-TH2 Cells That Promote B Cells: Follicular T Helper Cells (TFH)

According to the classic TH1/TH2 classification, TH1 is primarily restricted to cellular responses and TH2 to humoral responses. However, in TH1 responses, IFN*γ* can also induce B cell class switching towards IgG2a antibodies, and TH1-related antibodies facilitate anti-intracellular pathogen responses by opsonization of infected cells. Moreover, mice deficient in TH2 development, as obtained using STAT6 knockout mice that are essentially lacking IL-4, were unable to produce IgE, which is critical for the immune response against helminthes; nonetheless, these mice did respond to infections by producing IgG1, IgM, and IgG2a [48]. Thus, there are helper cells that are distinct from TH2 cells and can help promote B cell responses.

In order to ascertain that TFH are distinct from TH1/TH2, mice that lack the major genes encoding T helper subsets TH1, TH2, and TH17 were studied, confirming that TFH are indeed a unique subset [49], which express unique phenotype markers. Interestingly, these markers help to understand some of the mechanisms by which TFH facilitate B cell responses in lymph nodes. Indeed, these cells were termed* follicular T helper cells* because of their location in the lymph node follicle [50, 51]. They can be identified by high expression levels of ICOS, CXCR5, and the CXCR5-inducer, BCL-6 [52, 53]. Expression of this particular set of genes further downregulates non-TFH gene transcription [54]. TFH require IL-6 for their differentiation, as well as IL-21, which is necessary for expression of BCL-6 [53, 55, 56]. While circulating activated helper T cells have but transient CXCR5 expression, TFH express CXCR5 in a sustained manner.

While T cells normally express CCR7 and are thus guided to the T zone in lymph nodes, B cells localize to B cell follicles via CXCR5 and its cognate chemokine CXCL13, which is released from germinal center stromal cells. Interestingly, TFH-like ICOS^+^ extrafollicular CD4^+^ cells were observed immediately outside germinal centers and had correlated with autoreactive antibody production [57, 58]. Indeed, pre-TFH may encounter B cells due to their upregulation of CXCR5 (following antigen presentation by dendritic cells (DC)), while activated B cells increase CCR7 expression, therefore enabling migration toward each other and facilitating an encounter between B cells and pre-TFH in the B-T cell border. There, B cells present the internalized and processed BCR-specific antigen to T helper cells. Since the TFH are already activated by DC with the same antigen and are activated in a CD80/86-CD28-dependent manner [49, 59], they may serve as a second validation step before mounting potentially hazardous antibody production. By this, the foreign origin of the antigen is further verified, as CD40L and ICOS are presented to the cognate B cell, which is dependent on these signals for optimal activation, isotype switching, and survival.

TFH in the germinal center can thus facilitate class switching from IgM to IgG1, IgG2a, or IgA, based primarily on cytokine secretion. There, B cells are further positively selected by TFH according to their affinity to antigens during somatic hypermutation [60, 61]. In addition, TFH also increase survival of B cells by secreting IL-21, which in turn upregulates BCL-6 in both the B cell and the TFH. Failure to regulate antibody production by TFH can result in systemic autoimmunity, since antibodies can be produced against self-antigens. In a study that examined a mouse model of systemic lupus erythematosus (SLE), autoantibody production correlated with the presence of TFH; accordingly, anti-dsDNA antibodies were mounted [61]. Under these settings, suppression of TFH cell differentiation by mouse strain crossing with either BCL-6^+/−^ or SAP^−/−^ had reduced the emergence of SLE-like features but resulted in germinal center formation defects and immunodeficiency disorders [61]. In addition, adoptive transfer of TFH resulted in increased level of germinal center formation and also correlated with an SLE-like phenotype in unimmunized naïve wild-type mice [62].

### 2.5. TH22: Mediators with Epithelial Barriers

IL-22 is a member of the IL-10 cytokine family, originally identified in activated T cells and considered a TH1-related cytokine. Soon after the description of TH17 cells, IL-22 was reported to be expressed by IL-17-producing ROR*γ*T-expressing cells and was accordingly associated with TH17 cells [63, 64]. Its receptor, IL-22R, consists of two chains, one is the IL-22 receptor 1, and the other is the IL-10 receptor 2, which is also a component of IL-10 receptor. However, IL-22 is* not* a ligand for IL-10 receptor.

IL-22R is absent from immune cells. It is expressed mainly by epithelial barriers such as skin, kidney, respiratory tract, and gastrointestinal tract [65]. On the other hand, only hematopoietic cells express IL-22; it is expressed by activated T cells and, to a lesser extent, by NK cells [66], CD8^+^ T cells [67], and *γδ*T cells [68]. Therefore, IL-22 appears to take part in the crosstalk between immune cells and nonimmune cells, particularly at important sites of mucosal immune barriers.

IL-22 was depicted both as an immune-regulatory mediator, as well as a pathogenic cytokine, as it displayed a correlation with severity of chronic inflammation and with skin autoimmune disorders [69, 70]. IL-22 is involved in airway inflammation [71], as well as protection from liver inflammation [72], activation of fibroblasts, and wound repair [73]; IL-22 is also involved in rheumatoid arthritis [74] and systemic sclerosis [75]. Human CCR6^+^CCR4^+^CXCR3^−^CCR10^+^ memory T cells, considered as TH17-enriched cells, have a subpopulation of cells that express IL-22 and IL-13, but not IL-17, IFN*γ*, or IL-4; thus, TH22 are distinct from TH1, TH2, and TH17 subpopulations. However, similar to TH17 cells, RORC and aryl hydrocarbon receptor (AHR) signaling both appear to be essential for TH22 accumulation [76]. Nonetheless, although the main source of IL-22 is indeed TH22 cells, it can be also found expressed in TH1 and TH17 cells together with IFN*γ* or IL-17, respectively [77].

The cutaneous association of TH22 cells is quite prominent. CD11c^+^ dendritic cells help polarize CD4^+^ cells into TH22 by secreting TNF*α* and IL-6, but it appears that, preferentially, cutaneous DCs, rather than monocyte-derived DCs, will induce TH22 differentiation (62) [77]. TH22 cells were also found to present a skin-homing molecule, cutaneous lymphocyte-associated antigen (CLA), in addition to the CCR6, CCR4, and CCR10 receptors [69, 77]. In vitro, TH22 cells promote wound healing processes in keratinocytes [78], and, in the skin of patients with inflammatory disorders, such as psoriasis, atopic eczema, and allergic contact dermatitis, TH22 were found to infiltrate into the epidermis and affect wound healing and tissue repair processes by secreting fibroblast growth factor (FGF).

### 2.6. TH9

Up until recently, IL-9 was defined as TH2 cytokine, secreted from mast cells and T cells, correlating with levels of IL-3, IL-4, and IL-5, and associated with antiparasitic responses [79]. IL-9 was found to correlate with disorders such as autoimmunity, allergy, viral and intracellular infections, cancer, and transplantations [79–81]. Later studies showed that IL-9 can also be found produced by Tregs, TH1, and TH17 cells [79]. Finally, a unique subset of cells specialized in IL-9 production has been identified, TH9 cells [82].

TGF*β*, which is practically sufficient to drive CD4^+^ toward iTreg differentiation (at low IL-6 conditions) or TH17 differentiation (at high IL-6 conditions), is also the cytokine that determines TH9 maturation. The major TH2 cytokine, IL-4, that was initially reported to synergize with TGF*β* in order to induce IL-9 in CD4^+^ cells [83] appears to be but a* second signal* for the generation of TH9 [82]. Indeed, it was found that while IL-4 is able to* inhibit* TGF*β*-induced of foxp3 expression IL-4-derived GATA-3 is not inhibited by TGF*β* [84]. In another study, TH9 was shown to be induced by IL-10 in an IL-4-independent manner [85]. The IL-4-inducible factor, interferon regulatory factor 4 (IRF4) initially identified with TH2 cells and then as a TH17 transcription factor, was shown to have a significant role in the differentiation of TH9 cells [81]; IRF4 represses T-bet and foxp3 in T cells [86].

IL-17E, also known as IL-25, is another important cytokine for the development of TH9 cells and for IL-9 production. In vivo, IL-25 was found to correlate with severity of an IL-9-related disease model, that is, mouse allergic lung inflammation [87]. Accordingly, TGF*β* and IL-4-derived TH9 cells upregulate the receptor for IL-25, IL-17RB, and IL-25 signaling inducing IL-9 expression in a TGF*β*-dependent IL-4-independent manner; by this, it is suggested that IL-25 may serve as an alternative synergic signal to TGF*β* towards TH9 development.

Other cytokines that augment TH9 differentiation include type I interferons, IL-1, and IL-33. Both IFN*γ* and IFN*γ* enhance TH9 differentiation through the induction of IL-21, which also promotes TH17 and TFH, while IFN*γ*, the prototypical TH1 cytokine, suppresses IL-9 production [29, 49, 83, 84]. IL-1 and IL-33 were shown to induce IL-9 production [88, 89], while IL-23 has an inhibitory effect on IL-9 levels [90].

Like TH1, TH2, TH17, and Treg cells, it became necessary to identify a master regulator gene for the TH9 subset; indeed, none of the major T helper transcription factors, that is, T-bet, GATA-3, foxp3, or ROR*γ*T, are expressed by TH9 cells. Moreover, a unique transcription factor, PU.1, was found to induce differentiation towards TH9 cells. Accordingly, overexpression of PU.1 promotes IL-9 transcription and is a prerequisite for TH9 differentiation both during in vitro stimulation and during the in vivo course of the allergic pulmonary inflammation model [91].

**Figure 2:**
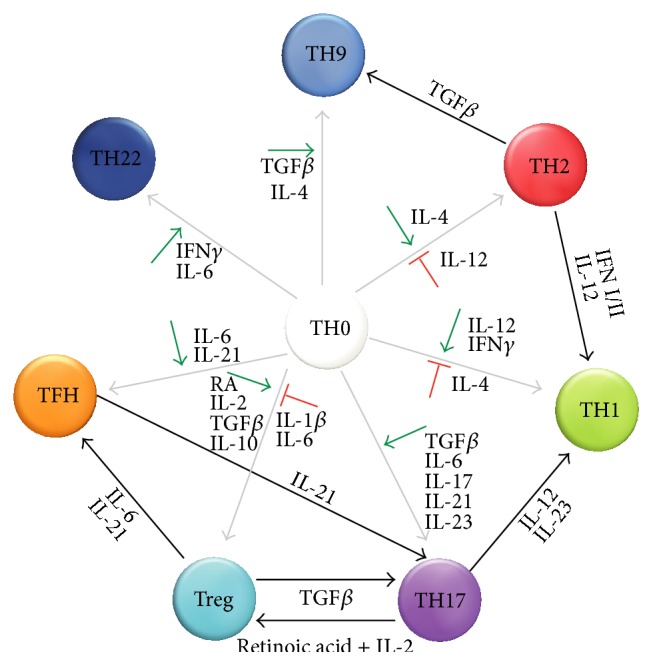
T helper (TH) cells differentiation and reprograming. TH0 cell can differentiate into specific TH subsets (*gray arrows*) under the influence of cytokines, which on the one hand can promote differentiation into a specific subset (*green arrows*) and on the other hand block differentiation towards a functionally-opposing subset (*red blocked arrows*). In addition, differentiated TH cells may be reprogramed to acquire a different TH phenotype (*black arrows*).

A summary of major helper T cell subsets and the cytokines that promote their development is presented in Figure 2.

## 3. Plasticity and Reprogramming of T Helper Phenotypes

In recent years, evidence is mounting to suggest that particular cytokine combinations may affect not only the differentiation of naïve TH0 cells but also that of differentiated T cells towards various helper subsets. This phenomenon is termed “T cells plasticity” and is quite significant considering that the adaptive response may switch from one type to another. To counter such plasticity, helper T cells secrete cytokines that* maintain* their subset phenotype. Thus, termination of an existing response and initiation of a new type of response are practically restricted due to this positive feedback loop.

Survival of a host is dependent upon an optimized and appropriate choice of strategy within adaptive immunity. This is most evident, for example, in the case of the response chosen towards intracellular and extracellular pathogens. Thus, the ability of differentiated T helpers to alter their phenotype when opposing signals develop is detrimental, and in situ effector cells require turning the subset master gene* off* while turning the transcription of a new master gene* on*.

Such plasticity is observed in the case of iTregs, as they turn into TH17: both types of cells depend on TGF*β*, either alone (for iTreg), or with IL-6 (for TH17). Thus, in a TGF*β*-rich environment, naïve T cells can differentiate into either one of the subsets according to the level of the acute phase cytokine, IL-6. This dichotomy in differentiation into either a pathogenic TH17 or a tissue protective Treg was demonstrated by Bettelli and colleagues, in an EAE mouse model [92]. Retinoic acid (RA) also possesses the capacity to alter the fate of a naïve T cell towards a helper T cell subset, as it preferentially promotes Tregs instead of TH17 cells; this is executed in an IL-2-dependent manner [93].

However, these two functionally antagonistic subsets are capable of switching phenotypes on-the-go: in a study by Xu et al., Treg cells not only contributed to the development of TH17 cells by secreting TGF*β* but had also themselves redifferentiated into TH17 in an IL-6-rich environment [94]. Yang et al. showed that iTreg and nTreg cells are able to redifferentiate into TH17 cells [95]. In another study using a foxp3 reporter mouse, it was demonstrated that ex-foxp3 positive cells are capable to re-differentiation towards IFN*γ*- or IL-17-producing T effector cells [96]. The group also evaluated re-differentiation of Tregs into effector cells carrying a TCR which recognizes islet *β* cell antigens in nonobese diabetic (NOD) mice and in BDC2.5-derived cells and concluded that Treg reprogramming is obtained also in autoimmune environment and may participate in pancreatic islet destruction. Another study used isolated TH17 from BDC2.5 mice, which were already known to exacerbated diabetes similar to TH1 cells in NOD/SCID mice [97].

Oral infection by* Toxoplasma gondii* (*T. gondii*) results in increased TH1 effector population, together with decreased numbers of Treg cells. This effect is possible both by the reduction in IL-2 levels, required for the survival of Tregs, and also due to the re-differentiation of Tregs into TH1 effector cells, which resulted in exacerbated gut pathogenesis [98].

Unlike other T helper cell subsets, TFH are suggested to have greater plasticity. TFH were shown to migrate within the germinal center, where they serve as helpers for B cells following adoptive transfer [60], and to display some relationship with TH17 subsets. For example, activation of TFH by ICOS, which in turn activates the transcription factor c-Maf, assists in maintaining TH17 clonal survival, most probably by the secretion of IL-21 [99]. IL-6, on the other hand, is required for the development of both TH17 and TFH [49].

Re-differentiation of Treg cells towards TFH was also demonstrated. Using foxp3-GFP reporter mice, transfer of foxp3^+^ cells into CD3*ε* ^−/−^ mice was shown to create novel germinal centers within the Peyer's patches, although, inexplicably, not in the spleen or LN; in these germinal centers, foxp3^+^ cells remained in the T zone, while cells which lost their foxp3 were localized in the B cell follicle [100]. These cells were found to have TFH phenotype.

Interestingly, plasticity between TH17 and TH1, two major effector subsets, was demonstrated to occur in the* absence* of TGF*β*: activation with IL-12 and IL-23 allowed for a switch from TH17 to IFN*γ*-expressing TH1 cells; these ex-TH17 TH1 phenotype was dependent on active STAT4 and T-bet [101].

TH2 cells appear to maintain the potential to redifferentiate into TH9 cells [82]. Veldhoen et al. cultured CD4^+^ cells with conditioned media from TH1, TH2, TH17, iTreg, and TH9 cells and observed that TH9 conditions did not differentiate naïve cells towards any subset other than TH9, and vice versa. Upon culturing IL-4-expressing TH2 cells, however, re-differentiation into TH9 was observed, at the expense of their TH2 characteristics, most probably due to stimulation with TGF*β*. These outcomes depict a capacity to reprogram TH9 and TH2 subtypes, both, incidentally, highly important in the immune response against helminths.

TH1 and TH2 are well known for their reciprocal inhibition of differentiation. Nonetheless, a study of T cell responses towards the well-known TH1 inductive virus, lymphocytic choriomeningitis virus (LCMV), challenges this dogma. The study shows that TH2-committed cells can alter their TH2 phenotype following the trigger of their TCR by LCMV, after induction with IL-12 and type I/II interferons [102]; experiments using adoptive transfer of TH2 cells into infected mice demonstrated clear TH2-to-TH1 reprogramming. These TH2 cells upregulated TH1-associated genes, however, with persisting TH2 genes.

## 4. Epigenetic Regulation of T Helper Subset Genes

How does a T helper cell decide between maintaining its phenotype or rather allowing changes in its profile? Some studies describe the presence of epigenetic gene regulation that can override a programmed subtype.

One of the many mechanisms of transcription control involves changes in chromatin accessibility without modifying the primary genetic code. This can be obtained by methylation of the DNA molecule on the cytosine of a CpG segment within the DNA sequence. A methylated CpG will silence genes that require silencing in a particular cell or tissue. Methylation pattern is inherited; thus, it is maintained in the differentiated phenotype of cells.

Another mechanism of transcription control involves compacting or releasing the nucleosomal structure at a regulatory site in a gene, profoundly affecting chromatin accessibility. Modifications of the histone tails can result in either heterochromatin (for repression) or euchromatin (for activation) of transcription. The “histone code” of cells is more flexible than DNA methylation and can be changed in order to reopen or shutdown transcription of genes based on relevant signals.

Polarization of stimulated T cells with activated NFAT1 is maintained by altering the accessibility of NFAT1 to the chromatin of* infg* or* il4* promoter in TH1 and TH2 cells, respectively [7]. Analysis of the* infg* gene shows that TH1 cells have H3k4me2-enriched histone modifications and increased CpG demethylation, thus allowing transcription of IFN*γ*; this is in contrast to TH2 cells, which display increased H3k27me3 (as opposed to H3k4me2) and excessive CpG methylation, counteracting the TH1 phenotype [103].

Global mapping of similar and other modifications, including H3K4me3 and H3K27me3, from T helpers of the TH1, TH2, TH17, nTreg, and iTreg subsets depicted some flexibility between subset-specific genes [104]. As expected, major cytokine promoters in each subset were indeed accessible to transcription in each appropriate cell type; for example, TH1, TH2, and TH17 were associated with H3K4me3 in* infg*,* il4*,* il17,* and* il21*, respectively. However, when examining transcription repression by H3k27me3, the situation appears to have become more complicated; while* infg* was rich in H3k27me3 in TH2 and TH17, this was not the case in nTregs. On the other hand,* il4* promoter was associated with H3k27me3 in TH1 cells, rather than in TH17 and Tregs. TH17 gene products, namely, IL-17 and IL-21, were more conclusive in as far as all other T helpers exhibited reduction in their transcription via H3K27me3.

Interestingly, at least one aspect of the mechanism behind* maintaining* plasticity of T helper subsets was elucidated by analysis of the epigenetic modifications of T helper master transcription factors. For example, H3k27me3 modification in TH1 and TH2 cells* silences* foxp3 but is less abundant in TH17 cells. In contrast, the gene for RORC did not contain H3K27me3 in iTregs, unlike in other subsets, and nTregs had both H3K4me3 and H3K27me3, indicating some plasticity in the distinction between TH17 and Tregs. Dnase I hypersensitivity (HS), an indicator of permissive chromatin obtained by H3K4me, is associated with* infg* loci or* il17a* and* il17f* in TH1 and TH17, respectively. However, TH17 cells stimulated with IL-12 exhibited higher levels of H3K4me in the* infg* loci, in addition to H3K27me3 repression of* il17a* and* il17f* loci. These histone-remodeling modifications are dependent on repression of RORC by H3K27me3 in the* rorc* locus, as mediated by T-bet and STAT4 [105].

TH1 and TH2 main transcription factor genes,* tbx21* and* Gata3*, were associated, as expected, with increased levels of H3K4me3 in the TH1 and TH2 accordingly. However, both had H3K4me3 and K3K27me3 in the opposite gene; this most probably allows the maintaining of a potential for a helper cell to establish a new phenotype. By reducing the abundance of K3K27me3, the promoter, now with only H3K4me3, may allow for a helper re-differentiation. This kind of epigenetic switch is known mainly to occur in multipotent stem cells that require the maintaining of genes in a repressed mode but poised for future activation [106].

## 5. The Implications of Elevated Acute Phase Protein, *α*1-Antitrypsin (AAT), on T Helper Cells

### 5.1. AAT Targets Non-T Cell Immunocytes and Modifies the Inflammatory Environment

AAT is a 52-kDa protein whose levels rise in the circulation during acute phase responses. It is primarily an anti-inflammatory agent, executing most of its activities by inhibition of inflammatory serine-proteases as reviewed in [107]. For example, neutrophils have been shown to be strongly affected by excess concentrations of AAT, as they rely on membrane neutrophil-elastase for extravasation and on degranulated elastase, cathepsin G, and proteinase-3 for further processing and activation of pivotal inflammatory molecules. Nonetheless, at high proximity, activated neutrophils will counter these activities of AAT by the release of oxidative radicals; these will readily neutralize the inhibitory capacity of AAT [108]. Indeed, oxidation of AAT by neutrophil-derived myeloperoxidase (MPO) leads to formation of IgA-AAT complexes, further reducing AAT activity to inhibit elastase [109]. In addition to oxidation by neutrophils, AAT may be oxidized by cigarette smoke. Although unable to inhibit elastase, oxidized AAT may gain inflammatory properties, leading to release of MCP-1, IL-6, IL-8, and TNF*α*, further allowing neutrophils and macrophages to spearhead towards their target tissues [110, 111]. Indeed, oxidized AAT was detected in Alzheimer's disease, heart failure, and premature rupture of the fetal membrane [112–114]. Despite reports attributing proinflammatory properties to oxidized AAT, Churg et al. have reported that oxidized AAT exerts anti-inflammatory activities, as it was able to reduce TNF*α* plasma levels in smoke-induced emphysema mouse model [115].

The oxidation of AAT may be reversible in the presence of reducing agents [116]. This may allow AAT to continue to protect tissues from excessive inflammation when it distances from the epicenter of a neutrophilic attack. Indeed, in the rare condition of genetic AAT deficiency, lung tissues of affected individuals exhibit an excessive number of neutrophils and subsequently the enzymatic degradation of alveolar walls, resulting in emphysema [117].

Similar to neutrophils, macrophages have been shown to be functionally altered by locally elevated levels of AAT. The predominant motif, again, is a change towards an anti-inflammatory environment: a sharp rise in inducible production of IL-10, a dramatic reduction in stimulated levels of IL-6, and, under some conditions, elevated release of TGF*β* [118–121]. Indeed, it has been postulated that AAT fits well in timing and spectrum of activities to act in the resolution phase of an inflammatory flare, a point in the immune response whereby the wound-healing aspects of innate immune cells reign. Yet, upon examining responses of DCs to elevated levels of AAT, the anti-inflammatory pretense of AAT changes; in its presence, DCs do not shut off all inflammatory responding genes. While they do express more IL-10 and less IL-6, they appear to display a semimature midrange costimulatory molecule profile and, most strikingly, a maintained expression of inflammation-stimulated CCR7 [122]. Accordingly, AAT-treated DCs migrate towards draining lymph nodes in a hastened manner, the outcome of which is an expansion of foxp3^+^ T helper cells, and the surfacing of a proposed immunological mechanism for the large number of reports on improved allograft survival during AAT treatment: beneficial changes in an EAE model [120], amelioration of disease markers in experimental RA [123], and profound reversal of the course of GvHD [124], ulcerative colitis [125], and type 1 autoimmune diabetes [126, 127]. Yet the most perplexing element within these attractive attributes of AAT is quite pivotal: AAT does not directly influence T cell responses.

The presence of any particular effect of AAT on T cells was examined in several studies. For example, human PBMCs were stimulated with various concentrations of IL-2 in the presence of AAT; then, IFN*γ* levels and cell proliferation were determined [128]. In this setup, AAT did not impair T cell proliferation nor did it alter IFN*γ* release. Similarly, mouse splenocytes were pretreated with AAT and stimulated with ConA. Again, proliferation and IFN*γ* release were unaffected by AAT. In contrast to lymphocytes, peritoneal macrophages that were stimulated with IFN*γ* and treated with AAT displayed reduction in nitric oxide release [129]. These setups combine innate and adaptive cells, limiting the ability to deduce the direct effect of AAT on T cell responses. Koulmanda et al. further examined the effects of AAT on T cells using CFSE labeled enriched T cells treated with anti-CD3 and anti-CD28 antibodies [126]. Again, AAT did not impair T cell proliferation or had any influence on acquisition of an activated phenotype. In as far as IL-2 levels are regarded, AAT was found not only to allow IL-2 release but also to enhance the levels of IL-2, as established in a coculture of OVA-stimulated DCs and OVA-responsive T cells [122]. Thus, it is by now assumed that the outcomes of AAT treatment, in which T cell subsets are altered in any way, is the result of changes experienced by innate cells that indirectly dictate to the T cells their preferential phenotype.

### 5.2. AAT and foxp3^+^ Regulatory T Helper Cells

The most notable* indirect* effect of AAT on T cells is the facilitation of foxp3^+^ Treg expansion. This attribute of AAT has been shown to persist across multiple immunological settings in which the progression of a given pathology is primarily antigen-driven. Treg expansion requires a particular cytokine milieu, that is, low levels of IL-1*β* [130], low levels of IL-6, and high levels of TGF*β* [92]. These trends in cytokine levels are afforded by AAT in a highly consistent manner both in vitro and in vivo. Moreover, another inflammation-inducible molecule that remains highly expressed in the presence of AAT is IL-1Ra, which is only elevated by AAT in cells exposed to an inflammatory signal [131]. Thus, by adding a layer of active inhibition on the IL-1 pathway, AAT breaks the vicious cycle of the IL-1-perpetuated inflammatory flare which, at least in part, was responsible for downplaying immune tolerance.

Koulmanda et al. have shown that AAT-treated NOD mice exhibit a reduction in T-bet and ROR*γ* transcription factors and that their expression of foxp3 was increased [126]. Accordingly, it was established that the autoimmune arm of pathology in the NOD mouse has been addressed by AAT as spontaneous diabetes was markedly diminished in treated NOD mouse cohorts, and the heightened response towards self-grafted autoreactive NOD islets was abrogated by preconditioning of the mice with AAT. Indeed, this latter outcome favors the possibility that a tolerant profile had been imprinted upon reactive T cell clones, perhaps so much as to suggest a tolerogenic antigen-specific memory profile.

### 5.3. The Significance of an Uninterrupted Stimulatory Expression of CCR7

AAT has been shown to promote semimature DCs [122]. Upon introduction of AAT to stimulated DCs, the cells display a reduction in costimulatory markers and an elevation in IL-10 production, while maintaining inducible inflammation-dependent membrane CCR7 levels. The latter indicates that AAT-induced semimature DCs can migrate towards the CCL19/21-releasing draining lymph node T zones. Indeed, the concept of a readily migrating semimature DC was important enough to challenge, as the current dogma of DC-directed T cell differentiation does require that the DC engages with a T cell in the lymph node. That an anti-inflammatory agent allows an array of inflammation-driven molecules to persist presents simultaneously as an indication and also as an enormous challenge in as far as the mechanism of action of AAT is regarded.

### 5.4. AAT Modifies T Helper-Related B Cell Responses

Treg population expansion may be also promoted by IL-10-producing AAT-derived B cells [132]. As reported by Mizrahi et al., LPS-stimulated B cells that were treated with AAT displayed a reduction in CD40, MHC class II, and CD86 surface levels, while at the same time they produced more IL-10 than untreated LPS-stimulated B cells. A similar trend was observed when, instead, B cells were stimulated with recombinant CD40L and IL-4. Interestingly, AAT was shown to increase Treg cell population in a B cell-dependent manner, as demonstrated in B cell-knockout mice; as described in the study, these mice were unable to mount an AAT-driven Treg expansion.

### 5.5. The T Helper-Predominant Xenoimmune Response and the Role of AAT-Modified Non-T Cells

An unexpected indirect effect of AAT on T cells came from combination studies. Based on the positive outcomes of islet allograft transplantation models using AAT therapy, Ashkenazi et al. examined the ability of AAT to prolong islet xenograft transplant survival [133]. Unfortunately, treatment with AAT alone failed to prolog xenograft survival even upon extending pretreatment duration and increasing its dose to the upper limit. Nonetheless, molecular profiles appeared to have changed for the better within explanted grafts, demonstrating more insulin transcripts and less inflammatory genes and important chemokines; thus, a combination therapy approach had followed.

When combining AAT treatment with a suboptimal dose of costimulatory blockade antibodies, an approach proven by itself to benefit xenograft survival, there appears to have been no significant change in outcomes; AAT plus costimulation blockade could not remove the immunological barrier that prevented an islet xenograft from being accepted by a diabetic host. However, when AAT was combined with temporal T cell depletion, yet another approach that holds an established background in literature and more importantly is already incorporated in clinical practice, xenografts exhibited significantly extended survival rates. Alone, removal of T cells by anti-CD3 or anti-CD4/CD8 depleting antibodies resulted in a mere temporary prolongation of xenograft survival. Once T cells repopulated the circulation, graft function was abruptly discontinued. However, repopulating T cells appeared to have responded to xenografts in a more favorable manner during simultaneous treatment with AAT. In fact, intragraft histology and gene expression outcomes revealed the strong presence of foxp3^+^ T cells in accepted grafts. These results suggest that AAT may modulate postdepletion repopulating T cells in favor of xenograft acceptance, by, at least in part, mediating an expansion in Tregs. The particular responding cells in this setup have yet to be determined, as is the question of whether these outcomes can be reproduced by adoptive transfer using AAT-preconditioned animals. In addition, it is not yet clear whether AAT is required to rise systemically for such positive outcomes or, conversely, the presence of AAT is necessary as a local entity in the graft microenvironment.

### 5.6. Evidence for the Utilization of Peripheral T Cell Plasticity by AAT

In order to depict whether AAT levels are required to be elevated in the circulation for its beneficial effects on islet *β* cell survival, a *β* cell line was generated that stably expresses human AAT (NIT-hAAT) [134]. Tested for its impact on lymphocytes, a mixed lymphocyte reaction (MLR) study was performed. According to study results, compared to control NIT-1 and NIT-vector cells, NIT-hAAT-primed lymphocytes and lymphocytes cocultured with NIT-hAAT cells displayed a significant reduction in IFN*γ* production and produced more IL-4, in effect skewing the TH1 response towards a TH2 response. In addition, lymphocyte proliferation rates when cocultured with NIT-hAAT cells were significantly reduced compared to those of lymphocytes cultured in the presence of control cells.

**Figure 3:**
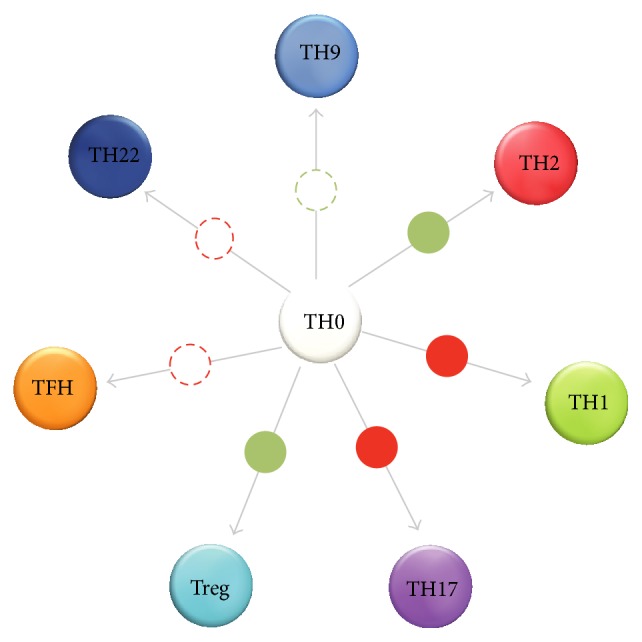
T cell differentiation in the presence of AAT. AAT has been shown to modulate cytokine levels with relevance to T cell differentiation. Circles superimposed on arrows indicate path facilitated by AAT (*green*) or suppressed by AAT (*red*); for each, a solid circle represents T cell-related experimental evidence and a dashed circle represent cytokine-related experimental evidence.

This intriguing system was then challenged in a transplantation model [134]. Mice that were grafted with NIT-hAAT cells displayed* reduction* in TH17 cell population size in the days following transplantation. These findings agree with the observed systemic effects of AAT, whereby human AAT-expressing mice exhibit reduced IL-17 levels and an expanded foxp3^+^ Tregs population in an EAE model [120]. Selected examples of the effects of AAT on TH subset differentiation are displayed in Figure 3.

## 6. Conclusion

Once one appreciates the large diversity and plasticity within the helper T cell arm of the immune system and the tight relation to local cytokines and the functions of local non-T cells, the possibility that a molecule alters practically every immune entity* but* T cells becomes less of a provocation. The fact that our liver generates 4–6-fold greater amounts of AAT during acute phase responses, at a time in which T cell activities must conquer pathogenic threats but in which their overenthusiasm might prove lethal to the host, fits the recent appreciation of the possibility that AAT acts by modifying responses as basic as inflammation, without interfering with the antigenic threat, the T cell axis. For this, the plethora of helper T cell subtypes appear to be superb partners for, based on local inflammatory cues, executing fine changes in the mounting of an untainted adaptive immune response.

There are vast aspects of helper T cell responses and their modification by AAT-conditioned environments that have yet to be studied. Beside the charting of the cytokine profile exerted by AAT under various conditions and its implication on the profile of helper T cells, there are many more interfaces to consider whereby AAT may alter the function of a T cell. Clinical studies that examine the prospect of AAT therapy for individuals who are not genetically deficient in AAT incorporate major findings recently established with regard to AAT and T cells, namely, recent onset type 1 autoimmune diabetes (NIH clinical trial registries NCT01183455, NCT01319331, NCT01183468, NCT02005848, NCT01304537, and NCT01661192) and treatment-resistant GvHD (NCT01523821).

Understanding in detail this novel niche between innate and adaptive immunity may allow for some novel and more precise designs of clinical trials for the optimal utilization of the readily available and safe for use molecule, AAT, without compromising T cell functionality.
